# Application of a novel RNA-protein interaction assay to develop inhibitors blocking RNA-binding of the HuR protein

**DOI:** 10.3389/fgene.2025.1549304

**Published:** 2025-03-05

**Authors:** Larissa Filcenkova, Annika Reisbitzer, Benjamin Philipp Joseph, Verena Weber, Paolo Carloni, Giulia Rossetti, Sybille Krauß

**Affiliations:** ^1^ Institute of Biology, Human Biology/Neurobiology, University of Siegen, Siegen, Germany; ^2^ Institute for Neuroscience and Medicine and Institute for Advanced Simulations (INM-9/IAS-5), Computational Biomedicine, Forschungszentrum Jülich, Jülich, Germany; ^3^ Faculty of Mathematics, Computer Science and Natural Sciences, RWTH Aachen, Aachen, Germany; ^4^ Department of Neurology, RWTH Aachen University, Aachen, Germany; ^5^ Juelich Supercomputing Center (JSC), Forschungszentrum Jülich, Jülich, Germany

**Keywords:** RNA-protein-binding, split-luciferase assay, NanoBiT, AU-rich elements, HuR inhibitor

## Abstract

RNA-protein interactions play an important regulatory role in several biological processes. For example, the RNA-binding protein HuR (human antigen R) binds to its target mRNAs and regulates their translation, stability, and subcellular localization. HuR is involved in the pathogenic processes of various diseases. Thus, small molecules blocking RNA-binding of HuR may be useful in a variety of diseases. Previously, we identified STK018404 as a small molecule targeting the HuR-RNA interaction. Based on this study we identified optimized compounds by exploiting combined structure-based and ligand-based computational approaches. To test a series of these compounds, we developed a novel readout system for the HuR-RNA interaction. Traditional methods to detect RNA-protein interaction come with some disadvantages: they require significant reagent optimization and may be difficult to optimize for weakly expressed RNA molecules. The readout often requires amplification. Thus, these methods are not well suited for quantitative analysis of RNA-protein interactions. To achieve an easy-to-perform, rapid, and robust detection of RNA-protein binding, we applied a split luciferase reporter system, to detect the interaction between HuR and its target RNA. We expressed one luciferase fragment as a fusion protein with HuR. The second luciferase fragment was Streptavidin-coated and coupled to a biotinylated RNA-oligo comprising an AU-rich HuR-binding element. The binding between HuR and its target RNA-oligo then allowed reconstitution of the functional luciferase that was detectable by luminescence. Using the split luciferase reporter system, we present here a series of optimized compounds that we developed.

## 1 Introduction

RNA-binding proteins and their association to their target RNAs are critically involved in a plethora of cellular processes including RNA processing, transport, and translation. Alterations of RNA-protein interactions impact multiple biological processes resulting in molecular phenotypes such as aberrant RNA splicing, RNA stability, RNA transport, and RNA translation. Aberrant RNA-protein interactions are increasingly recognized as critical determinants of neurological diseases. Mechanisms affecting the RNA-binding proteins, which are compromised in neurological disorders, include, for example, deregulated splicing (Schilling et al., 2016), deregulated RNA stability (Borgonetti et al., 2021), or aberrant translation (Griesche et al., 2016a; Krauss et al., 2013). These mechanisms affect neuronal function highlighting the susceptibility of neurons to aberrant RNA-protein interactions. Thus, emerging therapeutic approaches aim to inhibit aberrant RNA-protein interactions.

The HuR (Human antigen R) protein, encoded by the *ELAVL1* (embryonic lethal and abnormal vision) gene, is such an RNA-binding protein. It has several functions and is implicated in various physiological processes and disease development (Hinman and Lou, 2008). For example, HuR regulates stability, translation and the intracellular transport of its target mRNAs, indicating its key role in post transcriptional gene regulation (Hinman and Lou, 2008). HuR recognizes its target RNAs via adenylate and uridylate (AU)-rich sequence elements (AREs). The HuR protein contains three RNA recognition motifs (RRM1–3) responsible for binding AREs in mRNA. RRM1 and RRM2 are located at the flexible N-terminus, separated by a short 10-amino-acid linker, while a longer hinge region (50–60 amino acids) connects RRM2 to RRM3. This hinge region includes a nucleo-cytoplasmic shuttling element that transports HuR between the nucleus and cytoplasm and is also linked to protein-protein interactions. RRM1 initiates HuR binding to AREs, enhancing RRM2’s affinity for AREs. Together, RRM1 and RRM2 anchor HuR to mRNA. Although less understood, RRM3 also plays a critical role in target mRNA binding (Rajasingh, 2015; Doller et al., 2007; Doller et al., 2008; Pabis et al., 2019; Wang et al., 2013). Deregulation of HuR has been linked to a range of diseases, including neurological disorders (Borgonetti et al., 2021), inflammatory diseases (Zhou et al., 2007), and cancer (Abdelmohsen and Gorospe, 2010). For instance, dysregulated HuR binds to the AU-rich motif of the mRNA of *Bcl-2*. The binding enhances stability as well as the translation of Bcl-2, an anti-apoptotic protein. This leads to enhanced cell survival and contribution to tumorigenesis (Ishimaru et al., 2009; Abdelmohsen et al., 2014). Additionally, HuR also binds and stabilizes mRNAs encoding pro-inflammatory cytokines, thereby enhancing their expression (Krishnamurthy et al., 2010; Fu et al., 2024). For example, in myocardial infarct models, knockdown of HuR leads to a reduction in pro-inflammatory cytokine expression (Krishnamurthy et al., 2010). The involvement of HuR in several diseases makes it a promising target for pharmaceutical research.

Recently we performed an *in silico* approach to develop a new class of HuR inhibitors, which we tested for their efficacy in an RNA-protein pull-down assay. With this screening approach we identified the molecule STK018404 (4-(2-(2,4,6-trioxotetrahydropyrimidin-5(2H)-ylidene)hydrazinyl)benzoate) as a promising HuR inhibitor that blocks the binding of HuR to its target RNA. With its unique chemical structure and small size, this inhibitor shows potential for the development of novel drugs (Joseph et al., 2023).

While this study yielded a novel HuR inhibitor, it is important to note that the RNA-protein pull-down method has certain limitations. The RNA pull-down is a commonly used technique that isolates specific RNA molecules along with their binding proteins. However, it is a semi-quantitative technique that can be prone to various sources of error. For example, one challenge is the potential for nonspecific protein binding to the matrix (such as agarose beads) used in the assay. Additionally, weak interactions may be undetectable (Yang et al., 2021).

To address these challenges, we aimed to establish a novel method to study RNA-protein interactions utilizing the NanoBiT^®^ Protein-Protein Interaction (PPI) Starter System. The NanoBiT system is a luminescence-based technology that detects molecular interactions through a split-luciferase design. It consists of two separate fragments of the NanoLuc luciferase enzyme: a small fragment (SmBiT) and a large fragment (LgBiT). These fragments have minimal activity when separated. When tagged to interacting proteins of interest, they can be brought together through the interaction, reconstituting an active luciferase enzyme. This reconstitution generates a bright luminescent signal, providing a quantitative and highly sensitive readout of the interaction in real time.

Here, we successfully used the NanoBiT system in an innovative approach to detect RNA-protein interaction by coupling HuR to the LgBiT and the HuR target RNA (an AU-rich RNA element) to SmBiT. Upon interaction between HuR and its target RNA, the reconstituted NanoLuc luciferase produced a luminescent signal.

Utilizing this novel RNA-protein interaction assay, we tested our previously identified HuR inhibitor STK018404 along with three newly designed HuR inhibitors. Our luminescent-based approach enabled analysis of the interactions, providing a reliable method to detect and measure the inhibitory effects.

## 2 Methods

### 2.1 Luciferase assay

A biotinylated RNA oligonucleotide containing the HuR-binding motif [biotin-TEG-5′-AUUUUUAUUUU-3′, (IDT Integrated DNA Technologies)] and a mutant AU-rich RNA oligonucleotide, in which the Us have been replaced by Cs were dissolved in RNA structure buffer (10 mM Tris, pH 7, 10 mM MgCl_2_, 100 mM KCl) to a final concentration of 100 μM. The RNA was incubated at 72°C for 10 min, then slowly cooled to room temperature. Next, 5 µL of streptavidin-tagged SmBiT (Promega) was combined with 5 µL of the RNA oligo in 300 µL of Buffer D (20 mM Tris, pH 7.9, 20% glycerol, 0.1 M KCl, 0.2 mM EDTA, and 0.5 mM DTT) containing RNase inhibitor (RiboLock) and protease inhibitor (ThermoFisher). These components were incubated for 1 h to ensure complex formation.

To create the LgBiT-HuR fusion protein, the HuR cDNA (obtained from the plasmid GFP-HuR, Addgene) was cloned into the p.BiT1.3-N [CMV/LgBiT/Hyg] Vector (Promega) using the restriction enzyme XbaI. The vector was then transfected into 2 × 10^6^ HEK293T cells to express the LgBiT-HuR fusion protein. After 48 h, the cells were harvested with a cell scraper. The cells were lysed in 1 mL of Buffer D containing an RNase inhibitor (RiboLock) and a protease inhibitor (Thermo Scientific) by sonication. After removing cell debris by centrifugation at 12,000 g for 10 min at 4°C, the protein lysate was analyzed by SDS-PAGE and western blot utilizing an anti-LgBiT (Promega), anti-GAPDH (CST) and anti-HuR antibody (SantaCruz) to control for proper expression of the LgBiT-HuR fusion protein (Supplementary Figure S1).

The protein concentration of the LgBiT-HuR lysate was measured in a BCA assay (ThermoFisher). In a white 96-well plate, 20 µL of LgBiT-HuR (40 μg/mL) was combined with 10 µL of RNA-oligo-SmBiT and 55 µL of Buffer D. Subsequently, 100 µM of one of the HuR inhibitor-compounds (STK117443, STK333452, STK597483, STK018404) were added to the respective wells. The plate was then covered and incubated on a plate shaker at 300 rpm for 60 min at room temperature. After incubation, 25 µL of reconstituted Nano-Glo Live Cell Reagent (Promega) – prepared by mixing 1 volume of Nano-Glo Live Cell Substrate with 19 volumes of Nano-Glo LCS Dilution Buffer–was added to each well. Luminescence was measured using a Tecan Reader at room temperature, with readings taken every 5 min for a total duration of 1 h.

### 2.2 Virtual screening

The details of the performed virtual screening can be found here (Joseph et al., 2023).

### 2.3 Molecular docking studies

To obtain the docking poses of the compounds in the HuR protein binding cleft, we used molecular docking. Small molecules were prepared via LigPrep and docked into the HuR protein structure (4ED5) using the Schrodinger 2022.2 Glide standard docking protocol. The center of the grid was defined by the coordinates (−34.665, −14.191, −15.841) and the default settings of the standard docking protocol were applied.

### 2.4 Molecular dynamics simulations

The stability of the docking poses was subsequently assessed through unbiased molecular dynamics simulations conducted using GROMACS 2021.7 (Abraham et al., 2015). Each HuR-compound complex was solvated in a triclinic box with an edge length of 20 Å using the TIP3P model for explicit water molecules. In all simulations the AMBER 12 force field for proteins and ions and periodic boundary conditions were applied. To neutralize the overall charge of the system and achieve a salt concentration of approximately 150 mM, an appropriate number of potassium and chloride counterions were added. The ligand’s topology was generated using the General AMBER Force Field 2 (GAFF2) (He et al., 2020), with its partial atomic charges determined via the AM1-BCC semiempirical method (Jakalian et al., 2002). The LINCS algorithm was employed to restrain hydrogen bonds (Hess et al., 1997). Long-range interactions were calculated using a particle-mesh Ewald method with a grid spacing of 1.2 Å, while short-range electrostatic and van der Waals interactions were managed with a distance cutoff of 12 Å. In all simulations, the protein and solvent were coupled to separate heat baths, and temperature control was achieved using the velocity-rescale method (Bussi et al., 2007), with pressure coupling implemented via the Parrinello-Rahman method (Parrinello and Rahman, 1981). Before initiating the molecular dynamics simulations, each system underwent minimization using the steepest descent algorithm. A gradual heating approach was then applied, gradually raising the temperature to 310 K over 2 ns, followed by equilibration in an NVT ensemble for 10 ns with position restraints on the protein. Further equilibration was performed in an NPT ensemble for an additional 10 ns without position restraints, followed by a 500 ns production run.

## 3 Results

**FIGURE 1:**
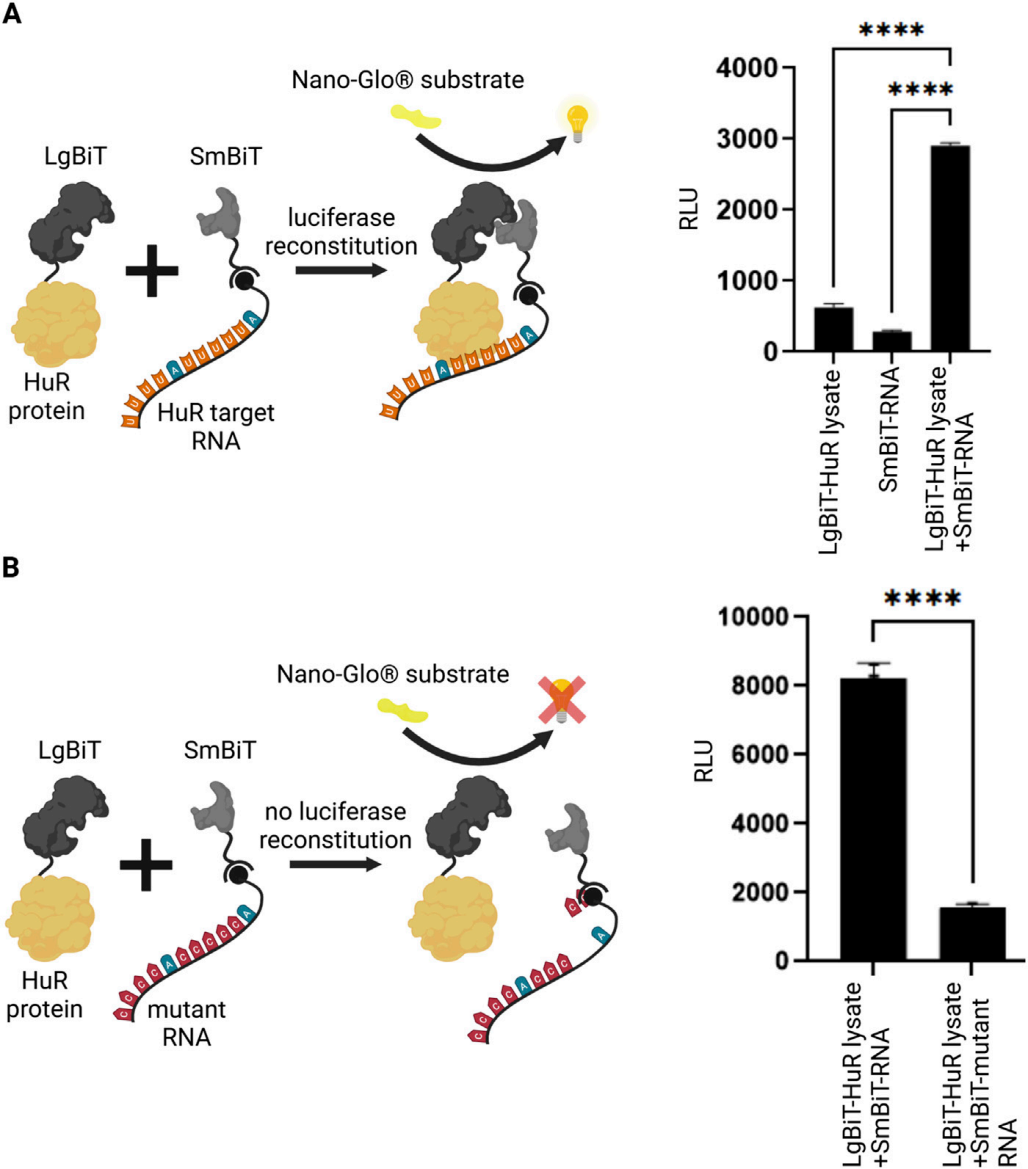
Split luciferase reporter assay for detection of RNA-protein interaction. **(A)** A split luciferase reporter assay was applied to detect RNA-protein interaction. The HuR protein (yellow) was fused to one luciferase subunit (LgBiT, dark grey) and the biotinylated HuR target-RNA (orange/blue) was coupled to the second streptavidin-tagged luciferase fragment (SmBiT, light grey). Upon interaction between HuR and its target-RNA, a functional luciferase is reconstituted which exhibits luminescence signal upon the addition of NanoGlo^®^ substrate (yellow). As negative controls, the LgBiT-HuR lysate without RNA-coupled SmBiT and RNA-coupled SmBiT without LgBiT-HuR were tested. Columns show the quantification of relative light unit (RLU) (mean value ± SEM, n = 3, p****<0.0001), after 50 min. **(B)** The same assay was performed using a mutant RNA, that has a significantly reduced binding affinity to HuR. In the absence of an interaction between HuR protein and the mutant RNA (red/blue), the luciferase fragments remain non-functional. Columns show the quantification of relative light unit (RLU) (mean value ± SEM, n = 3, p****<0.0001), after 50min. (Schematics created with Biorender.com).

In an initial set of experiments, we aimed to test if the interaction between HuR and its target RNA is detectable using the NanoBiT System. We expressed the HuR protein tagged to the LgBiT and coupled the HuR target RNA in form of a biotinylated AU-rich RNA oligo to streptavidin-conjugated SmBiT. LgBiT-HuR lysate and the SmBiT-tagged RNA were then co-incubated and analyzed in a luciferase reporter assay. Upon interaction between HuR and its target RNA, the reconstituted NanoLuc luciferase produced a luminescent signal. The negative controls in which the LgBiT-HuR lysate was incubated without SmBiT-RNA or the SmBiT-tagged RNA was incubated without LgBiT-HuR gave a significantly lower background signal (Figure 1A). Similarly, incubation of SmBiT-tagged RNA with extracts of untransfected HEK293T cells gave no significant luciferase signal (Supplementary Figure S2). This experiment shows that detection of the HuR-RNA-interaction is possible using this system. As another control for this assay, we compared the interaction between HuR and its target RNA to an experiment with a mutant AU-rich RNA element. As expected, a significantly lower signal was detected when using the mutant RNA (Figure 1B).

**FIGURE 2:**
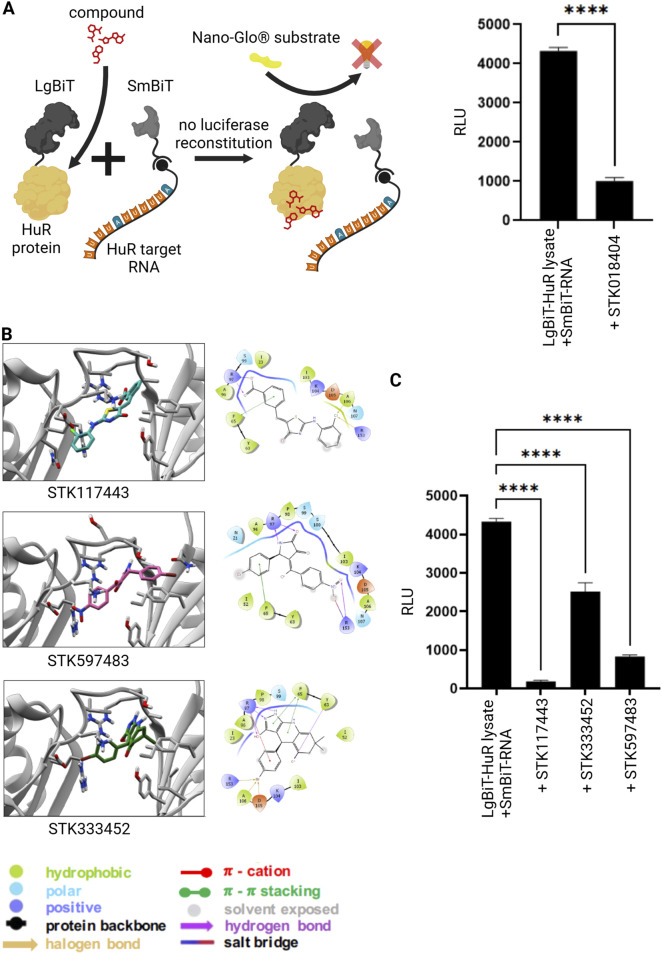
Inhibition of RNA-protein interaction via small molecules. **(A)** Split luciferase reporter assay detecting interaction between the HuR protein (grey) and its target RNA as described in Figure 1. In the presence of a previously published HuR inhibitor (red) (Joseph et al., 2023) the interaction between HuR and its target-RNA is blocked and the luciferase fragments remain non-functional despite substrate (NanoGlo^®^ substrate, yellow) availability. Columns show the quantification of relative light unit (RLU) (mean value ± SEM, n = 3, p****<0.0001), after 50 min. **(B)** Bidimensional view and ligand interaction diagram of STK117443, STK597483 and STK333452. **(C)** A split luciferase assay detecting the interaction between HuR and its target RNA was performed as described above in the presence of the novel HuR inhibitors STK117443 (+STK117443, 100µM, p****<0.0001), STK333452 (+STK333452, 100 µM, p****<0.0001), STK597483 (+STK597483, 100 µM, p****<0.0001), after 60 min at RT. (Schematics created with Biorender.com).

In our previous studies, we identified STK018404 as HuR inhibitor that can block HuR-RNA interaction (Joseph et al., 2023). Thus, we applied this compound in our novel NanoLuc luciferase assay to monitor HuR-RNA binding. Indeed, addition of STK018404 to the experiment resulted in a significantly reduced luminescent signal (Figure 2A), showing that this assay is a valuable tool to test HuR-inhibitors. In our previous work we applied structure-based virtual screening to generate an enlarged chemical space of molecules potentially targeting HuR and impairing RNA binding (Joseph et al., 2023). Among the candidates selected for experimental validation, STK018404 stood out as one of the few inhibitors with a dose-dependent effect on RNA binding against the full-length HuR protein. Notably, it features a scaffold distinct from previously known inhibitors (Supplementary Figure S3). Docking calculations suggested that STK018404 binds to the transient cleft, which also accommodates cognate mRNA nucleobases and other small ligands (Nasti et al., 2017). In one predicted binding pose, the compound interacts with Arg97, Phe65, Tyr63, and Lys104 (Supplementary Figure S3A, B). In another pose, which achieved an equivalent docking score (Supplementary Table S1), it forms two hydrogen bonds with Arg97 and both a hydrogen bond and a salt bridge with Lys92 (Supplementary Figure S3C). This latter pose was prioritized during the screening phase of our earlier work (Doller et al., 2008).

Independently of the binding pose, some of these residues (Arg97, Phe65, Tyr63) are also known to interact with a variety of other ligands, including Hyperoside, Novobiocin and 7-hydroxymatairesinol (Nasti et al., 2017; Vasile et al., 2018), as well as uracil nucleotides of the RNA (U8 and U9 in PDB 4ED5) (Wang et al., 2013). To improve the translational outcome of our approach, we focused here on other three molecules, identified in the same screening, STK117443, STK597483, STK333452, that also display a very different scaffold from previously known inhibitors and are shown here to efficiently block the HuR-RNA interaction.

By analyzing their docking poses, we noticed that all three interact with Arg97 (bifurcated hydrogen bond) and Phe65 (pi-stacking interaction). STK117443 additionally interacts with Asn25, while STK597483 and STK333452 interact with Arg153. Interestingly, while STK333452 appears to establish the higher number of interactions (in addition to the above-mentioned, it also interacts with Tyr63, Asp105 and Ala106), it is also more exposed to the solvent and only interacts with the hinge region and its adjacent residues. Out of the four HuR inhibitors identified by our structure-based virtual screening, STK117443 spans the biggest part of the binding cleft and might therefore most effectively hamper RNA binding.

After performing MD simulation on all four complexes, we found that throughout 500 ns the binding cleft formed by the two RNA recognition motifs RRM1 and RRM2 is preserved in the presence of each ligand. Interestingly, it also closes-up around the ligands, causing the orientation of both RRMs to slightly shift with respect to each other, shortening the distance between them. We further observed that the binding poses of the compounds STK117443 and STK597483 are dynamic and their position with respect to the protein adapts upon alteration of the binding cleft (Figure 2B; Supplementary Figure S4, B). Nevertheless, both compounds were shown to form persistent interactions with the HuR protein: Both molecules form hydrogen bonds with the arginine residues 97 and 153. However, STK597483 contacts both residues in parallel through the keto, hydroxyl and amide groups, while the benzoic acid moiety of STK117443 only forms a hydrogen bond with either of these arginine residues at a time (Supplementary Figure S5). Solely, STK333452 (Figure 2B) seems to be stable in the binding pose spanning the hinge region of the HuR protein as predicted by molecular docking (Supplementary Figure S4C). Concerning our previously published molecule STK018404 (Joseph et al., 2023), as said above, two different binding modes were found (Supplementary Figures S4A,B). Notably, molecular dynamics simulations reveal that one binding pose (Supplementary Figure S3C) converges to the other binding pose (Supplementary Figure S3B already during equilibration.

Finally, we tested these newly designed compounds for their efficiency to block the HuR-RNA interaction both in an RNA pull-down experiment (Supplementary Figure S6) as well as in our novel NanoLuc luciferase assay (Figure 2C). Strikingly, all three novel inhibitors showed significant effects on the HuR-RNA binding.

## 4 Discussion

RNA-binding proteins play crucial roles in the post-transcriptional regulation of gene expression, impacting several cellular processes such as the maturation, modification, transport and degradation of both coding and non-coding RNA molecules (Gerstberger et al., 2014). Alterations of RNA-protein interactions are increasingly recognized as critical determinants of neurological diseases. Thus, novel therapeutic approaches aim to block aberrant RNA-protein interactions. This can be achieved, for example, by small molecules that block RNA-protein binding (Joseph et al., 2023; Matthes et al., 2018). The development of such inhibitors requires read-out techniques that quantitively and accurately detect RNA-protein interactions.

In this study we developed a method that allows an easy to handle, fast, and robust detection of the RNA-protein binding. By adapting Promega’s NanoBiT PPI Starter System, we achieved a reliable method to detect RNA-protein interaction that is much better suited for *in vitro* drug screening assays than common methods. Commonly used methods for studying RNA-protein interactions like electrophoretic mobility shift assays (EMSA), RNA pull-down assays, RNA-immunoprecipitation (RIP), RNase protection assays (RPA), or colocalization studies using RNA-Fluorescent *in situ* hybridization (FISH) come with some disadvantages. Some of these techniques require cross-linking, which makes it difficult to detect binding kinetics, other techniques are difficult to optimize for rare RNA molecules and may lack the necessary sensitivity. Moreover, most of these techniques provide qualitative, but only semi-quantitative information, which makes it difficult to study dose-dependent effects of molecules inhibiting RNA-protein interactions. For example, RNA pull-down has the potential for nonspecific protein binding to the matrix (such as agarose beads) used in the assay. Additionally, weak interactions may be undetectable (Yang et al., 2021). With RNA pull-down we previously identified an HuR-inhibitor (Joseph et al., 2023). However, due its semiquantitative nature this approach is not ideal for intense dose-response of efficacy experiments. Similar challenges are encountered with other common techniques like EMSA (Zhao et al., 2020), RIP (Griesche et al., 2016b), or RNase assisted chromatography (Krauss et al., 2013). Taken together, each of these methods may show limitations in specificity, reproducibility, and sensitivity. Thus, these methods are not ideal to quantitatively study RNA-protein interactions, which is of utmost importance, for example, in drug screening assays, in which the efficacy of an inhibitor should be measured. In our study using the NanoBiT system, we were able to develop an easy-to-handle, reliable, robust method to detect RNA-protein interaction that is much better suited for *in vitro* drug screening assays than these common methods.

This is the first report of application of the NanoBiT system for *in vitro* analysis of RNA-protein interactions. While in a recent study the NanoBiT PPI Starter System has been used to investigate RNA-protein interactions *in cellulo*, via the so-called RNA interaction with protein-mediated complementation assay (RiPCA) (Rosenblum and Garner, 2022), our application is different. RiPCA enables detection of intracellular RNA-protein interactions by combining biorthogonal chemistry and split-luciferase technology. In brief, a stable cell line expressing the SmBiT fused to a HaloTag, an engineered dehalogenase that covalently binds to chloroalkane-containing ligands, is used. These cells are then transfected with a plasmid encoding the RNA-binding protein tagged to the LgBiT and a chloroalkane modified RNA probe. This RNA-probe will then be conjugated to SmBiT-HaloTag. Upon interaction between this SmBiT-labeled RNA and LgBiT-tagged RNA-binding-protein the NanoLuc will be reconstituted, generating a chemiluminescent upon treatment with the luciferase substrate. In our work, we describe a complementary *in vitro* approach that offers an alternative option for the analysis of RNA-protein interactions. This method is fast and easy to perform due to the straightforward biotin-streptavidin interaction for RNA tagging. The *in vitro* setup reduces variability associated with cellular systems and allows a quick, high-throughput screening of RNA-protein interactions, making it suitable for initial compound screening. Nevertheless, transitioning from *in vitro* to an *in cellulo* assay remains a crucial next step to obtain information regarding potential candidates.

In conclusion, given the growing interest in studying RNA-binding proteins and their role in diverse diseases, reliable and easy-to-handle methods to detect RNA-protein interactions are required. We present here a novel, robust method to detect RNA-protein interactions. Using this novel tool, we further tested a set of HuR inhibitors. The dysregulated HuR protein plays a major role in neurodegenerative diseases (Borgonetti et al., 2021), cancer (Zhou et al., 2007) or inflammatory diseases (Abdelmohsen and Gorospe, 2010). Therefore, HuR inhibition holds potential to restore cellular homeostasis in disease states. Blocking HuR may lead to downregulation of pro-inflammatory cytokines (Krishnamurthy et al., 2010), oncogenic factors (Zou et al., 2022) or proteins involved in cellular stress responses (Yang et al., 2024). Future experiments will aim at transferring our results to intracellular assays to further broaden the field of application of our new HuR inhibitors.

## Data Availability

The original contributions presented in the study are included in the article/Supplementary Material, further inquiries can be directed to the corresponding authors.
