# Supplementary material for: Application of a novel RNA-protein interaction assay to develop inhibitors blocking RNA-binding of the HuR protein
Source: Front Genet. 2025 Mar 5;16:1549304. doi: 10.3389/fgene.2025.1549304 (PMC11921777; doi:10.3389/fgene.2025.1549304)
Supplement: Supplementary file 1 [file DataSheet1.docx]

Supplementary Material

# Supplementary Data

**Methods: RNA Pull-down**

A biotinylated RNA oligonucleotide containing the HuR-binding motif (biotin-TEG-5'-AUUUUUAUUUU-3’, (IDT Integrated DNA Technologies)) was dissolved in RNA structure buffer (10 mM Tris, pH 7, 10 mM MgCl₂, 100 mM KCl) to a final concentration of 100 μM. The RNA was incubated at 72°C for 10 minutes, then slowly cooled to room temperature. To coat streptavidin-agarose beads (Sigma, St. Louis, MO, USA) with the RNA, 300 pmol of RNA oligo were added to 60 μL of beads in 300 μL of Buffer D (20 mM Tris, pH 7.9, 20% glycerol, 0.1 M KCl, 0.2 mM EDTA, and 0.5 mM DTT) and incubated for 30 minutes at room temperature.

To obtain HuR-containing cell extracts, 4 x 10⁶ HEK293T cells were seeded in a 150 cm² flask, cultured for 48 hours, and harvested with a cell scraper. The cells were lysed in 1 mL of Buffer D containing an RNase inhibitor (RiboLock) and a protease inhibitor (Thermo Scientific) by sonication. After removing cell debris by centrifugation at 12,000 g for 10 minutes at 4°C, the lysate was pre-cleared and incubated with the RNA-coated beads overnight at 4°C, with or without 100 µM of the compounds of interest.

After washing three times, the RNA-bound proteins were eluted by boiling the beads with 2x Magic Mix (48% Urea, 15mM Tris-HCl, pH 7.5, 8.7% Glycerine, 1% SDS, 0.004% Bromphenolblue, 143mM Mercaptoethanol) for 5 minutes at 95°C. The RNA-bound proteins were analyzed by western blot utilizing an anti-HuR antibody (Santa Cruz, Dallas, TX, USA, SC5261) to detect HuR.

# Supplementary Figures

**
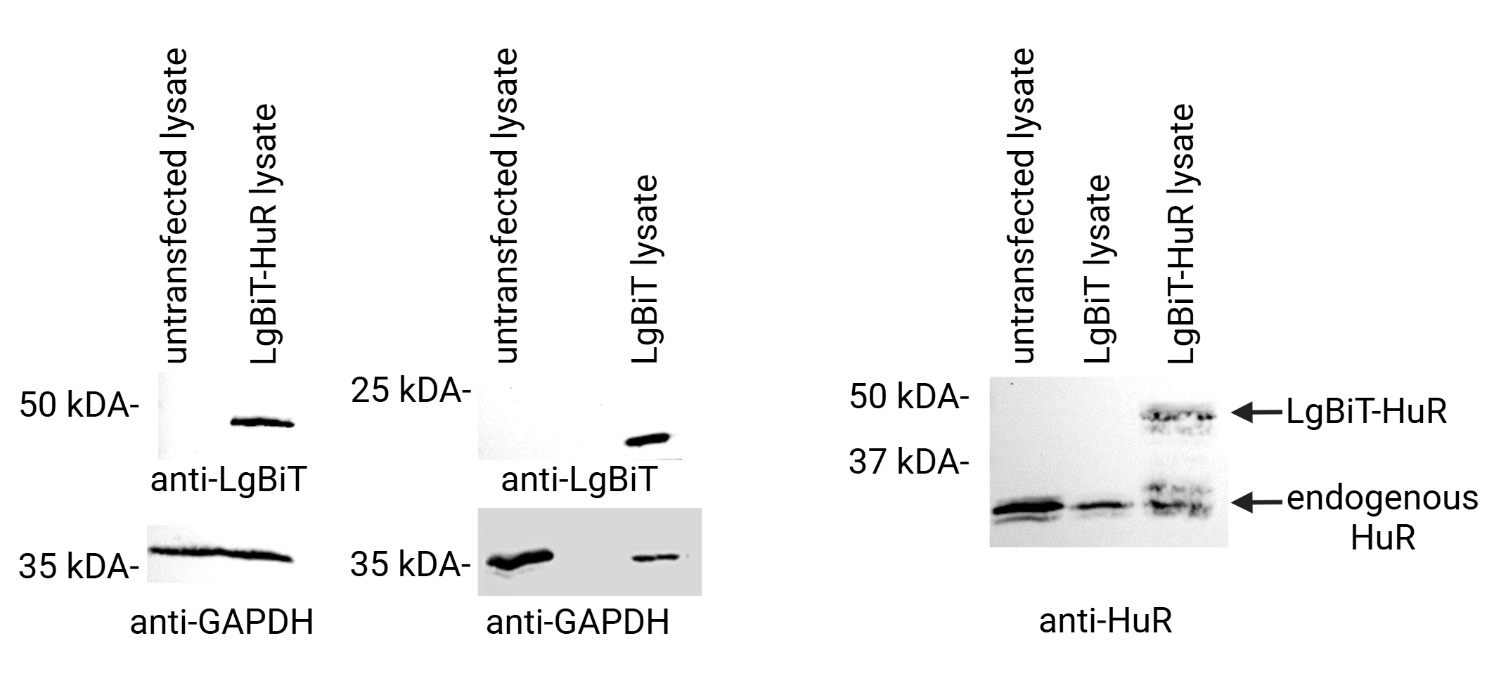
**

**Supplementary Figure S1: Transfection control of LgBiT alone and LgBiT-HuR by western blot.**

Untransfected HEK293T protein lysate (untransfected lysate), protein lysate from HEK293T cells transfected with p.BIT1.3-N (LgBiT lysate) and protein lysate from HEK293T cells transfected with p.BIT1.3-HuR (LgBiT-HuR) detected on a western blot using anti-LgBiT, anti-GAPDH and anti-HuR antibodies.


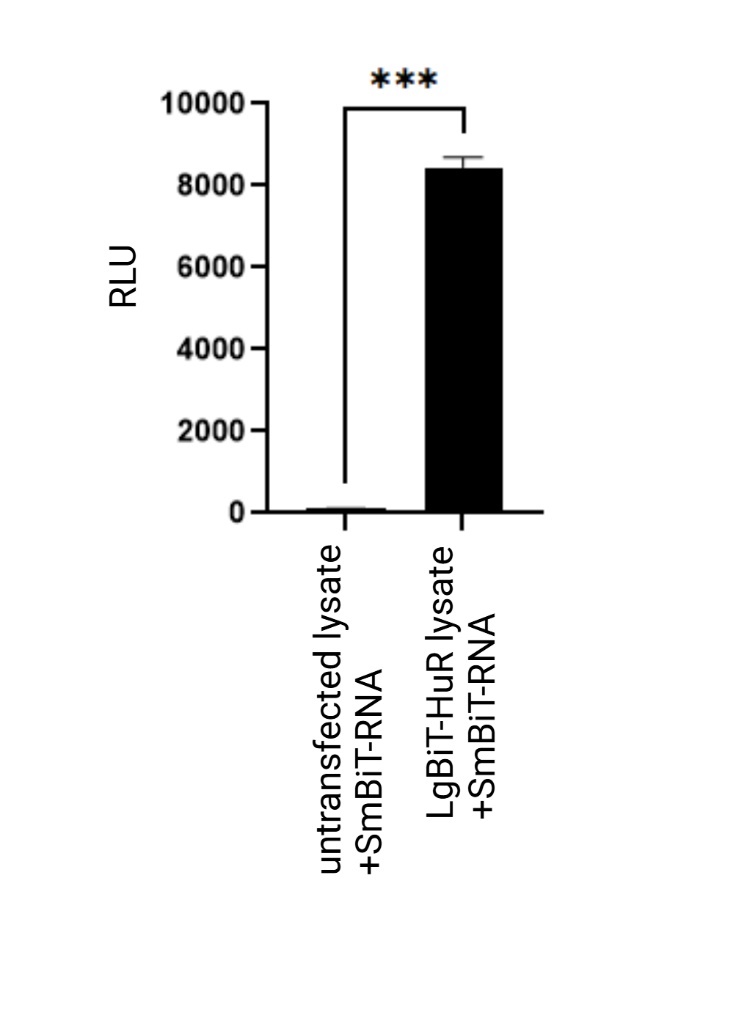


**Supplementary Figure S2: Negative control for the split luciferase reporter assay.**

A negative control of the split luciferase reporter assay detecting interaction between the HuR protein and its target RNA described in Figure 1. Upon interaction between HuR and its target-RNA, a functional luciferase is reconstituted which exhibits luminescence signal upon the addition of NanoGlo^®^ substrate. As negative control, RNA-coupled SmBiT with non-transfected HEK293T protein lysate was tested. Columns show the quantification of relative light unit (RLU) (mean value +/- SEM, n=3, p***<0.001), after 50min.


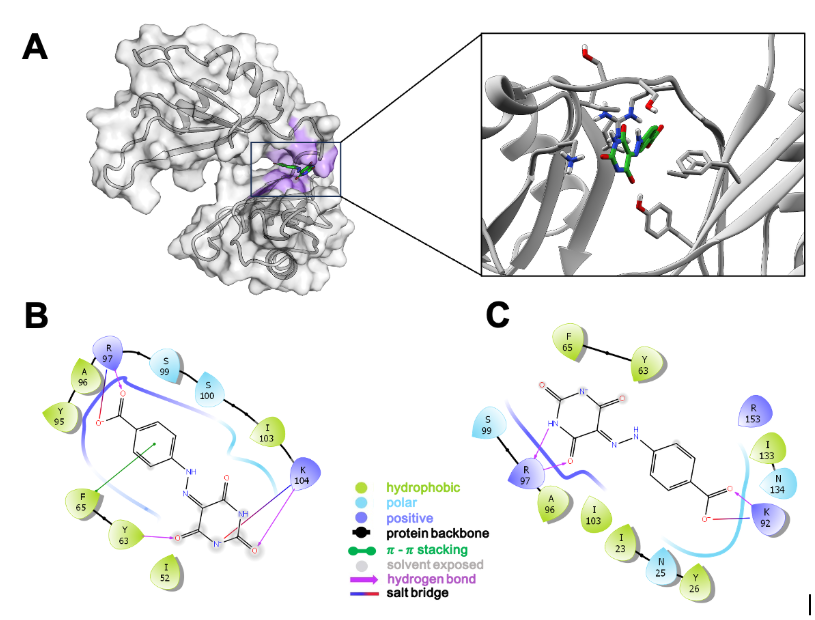


**Supplementary Figure S3: Binding pose of STK018404 1 in tridimensional and bidimensional view.**

In (A) the protein is in cartoon representation with a protein surface shadow, while the ligand is in licorice representation. The binding site is highlighted in violet. A zoom in of the binding site is offered in the insert. (B) The corresponding 2D interaction diagram shows the interaction of the ligand with the binding site residues. The color of the residues indicates their physicochemical properties hydrophobicity (green), positive charge (violet) and polarity (cyan), whereas the colorful thick line surrounding the ligand represents the binding cavity, with the color property being the same as for the interacting residues. Gaps in this line indicating solvent exposure of the ligand. Black lines connecting consecutive residues represent the protein backbone. (C) The 2D interaction diagram shows the previously identified binding pose of STK018404.


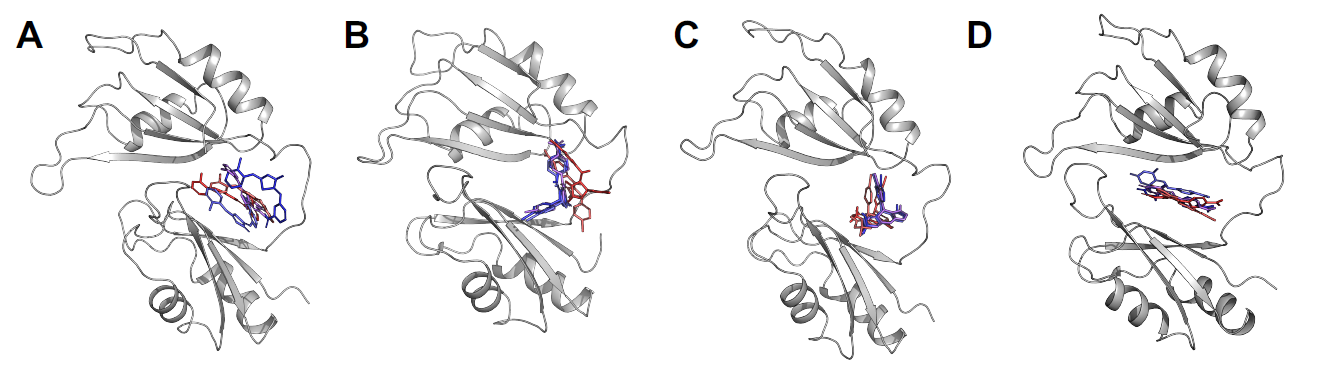


**Supplementary Figure S4**: **Simulation of the HuR molecular dynamics.**

HuR protein (grey) structure after 500 ns of unbiased molecular dynamics simulation; (A) in complex with STK117443 after 100 ns (red), 200 ns (orange), 300 ns (violet), 400 ns (light blue) and 500 ns (dark blue); (B) in complex with STK597483 (C) in complex with STK333452 (D) in complex with STK018404


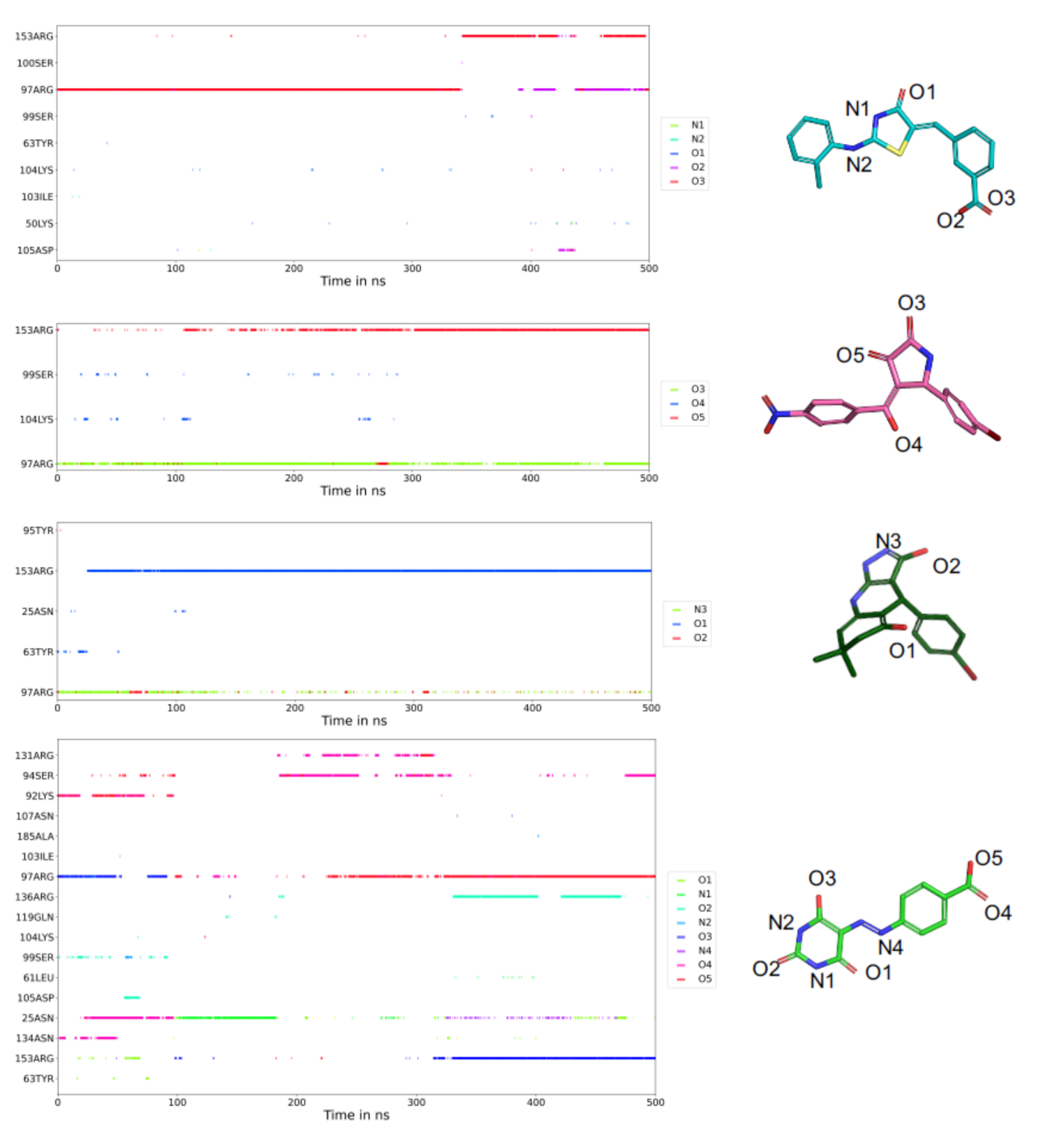


**Supplementary Figure S5: Computational 3D molecular representation of hydrogen bonds of the tested compounds.**

The occurrence of the hydrogen bonds established by each molecule within the cleft are reported as a function of the simulated time. The hydrogen bond donors/acceptors here considered are labeled on the nearby 3D molecular representation.


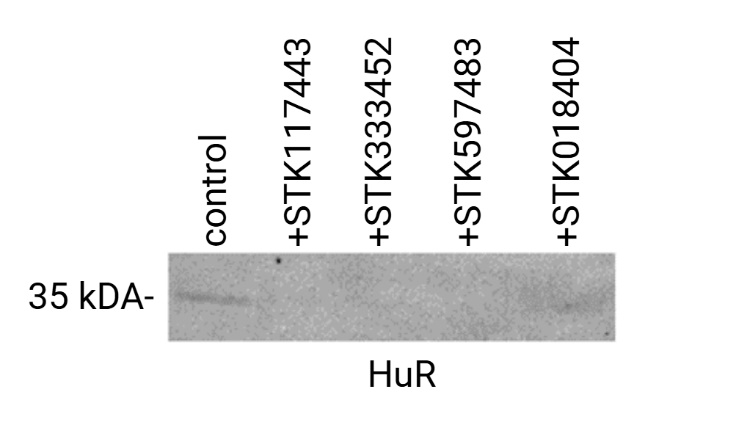


**Supplementary Figure S6:** **RNA pull-down of the HuR protein via AU-rich RNA motif.**

RNA pull-down non-treated (control) and with 100 µM compound treatment (+STK117443, +STK333452, +STK597483, +STK018404) incubated overnight at 4°C. Biotin-labeled RNA oligo was immobilized on agarose beads and RNA-bound proteins were isolated. Western blot was used to identify HuR (Anti-HuR, Santa Cruz Biotechnology).

**Supplementary Table S1:** **Docking scores of the tested compounds.**

Docking scores as calculated by Schrodinger Glide Version 2022.1 Friesner et al., 2004; Friesner et al., 2006 of the four molecules are reported.

|  | Glide Score standard protocol | Glide Score extended protocol |
| --- | --- | --- |
| STK117443 | -6.17 | -7.40 |
| STK597483 | -7.88 | -9.03 |
| STK333452 | -7.42 | -5.94 |
| STK018404 | -7.06 | -7.18 |
